# Academic motivation at early ages: Spanish validation of the Elementary School Motivation Scale (ESMS-E)

**DOI:** 10.3389/fpsyg.2022.980434

**Published:** 2022-12-07

**Authors:** Marta Ramos, Raquel De Sixte, Álvaro Jáñez, Javier Rosales

**Affiliations:** Developmental and Educational Psychology, Universidad de Salamanca, Salamanca, Spain

**Keywords:** Self-Determination Theory, early ages, reading, mathematics, writing

## Abstract

The Elementary School Motivation Scale (ESMS) is the only validated and adapted instrument to assess motivation in early ages and in specific domains using the Self-Determination Theory. The present study aims to validate the ESMS in Spanish population (ESMS-E). To this end, 1,190 students from the first half of Elementary education (6–10 years old) filled in the ESMS-E. To translate the instrument the back translation method was used. Internal consistency was assessed through composite reliability (CR), correlations among the dimensions and Confirmatory Factor Analysis (CFA) were used to analyze the theoretical structure proposed by the original instrument. Results showed optimal internal consistency in the different dimensions (CR = 0.701–0.901) and showed a great fit for the model (RMSEA = 0.064, CFI = 0.929, TLI = 0.913), confirming the original model with nine dimensions and the self-determination continuum. The ESMS-E is valid and reliable in the Spanish version. This validation offers a tool for researchers interested in exploring the motives that drive students in early stages in relation to specific learning domains (i.e., reading, writing and mathematics).

## Introduction

The interest in studying motivation in early ages and its role on performance in specific domains is increasing lately (e.g., Garon-Carrier et al., 2016; Schiefele et al., 2016; Mercader et al., 2017; Silinskas and Kikas, 2019; De Sixte et al., 2020; Kanonire et al., 2020). To this end, the Self-Determination Theory (SDT; see Deci and Ryan, 1985; Ryan and Deci, 2017, 2019, 2020) offers a solid framework to study motivation in educational contexts (Bureau et al., 2021; Howard et al., 2021) from an early age. Proof of this is the proposal of scales such as the ESMS (Guay et al., 2010) that allows the exploration of some of the types of motivation considered in the continuum proposed by SDT. Specifically, through this scale it is possible to study intrinsic and extrinsic motivation, the latter in two specific typologies: identified and controlled. It also enables this exploration to be carried out in three specific domains: reading, mathematics and writing, which is why it is so important to consider its validation in the Spanish population, given the research possibilities offered by an instrument of these characteristics. In this sense, countries such as Russia (Kanonire et al., 2020) or Canada (Guay et al., 2010) have this tool already validated, but Spain does not. Therefore, the present study aims to validate the ESMS in Spanish population (ESMS-E).

## Literature review

According to SDT, motivation is defined in terms of the reasons underlying a behavior, which may vary according to their degree of self-determination (i.e. autonomous or controlled; Ryan and Deci, 2019; Howard et al., 2021).

Specifically, SDT distinguishes between intrinsic and extrinsic motivation. The former refers to activities that are undertaken for their own interest or enjoyment, because they are satisfying in themselves (Deci and Ryan, 2000; Ryan and Deci, 2020). Therefore, the reasons for engaging in learning are linked to the enjoyment and interest in performing those tasks. On the other hand, when the reasons for action are instrumental, in the pursuit of an outcome independent of the activity itself, the motivation is defined as extrinsic (Deci and Ryan, 2000; Ryan and Deci, 2020).

Unlike intrinsic motivation, which is always self-determined, extrinsic motivation has different levels of self-determination. From lowest to highest self-determination: external regulation, introjected regulation, identified regulation and integrated regulation (Howard et al., 2017, 2020, 2021; Ryan and Deci, 2020). While external regulation seeks rewards or avoidance of punishment, introjected regulation seeks approval from self and others through feelings of pride, obligation and guilt (De Naeghel et al., 2012; Ryan and Deci, 2020; Howard et al., 2021). When regulation is identified, we engage in an activity simply because of perceived personal values, regardless of the potential satisfaction they may bring, and integrated regulation occurs when our behavior is perceived as part of our own identity. In this way, not only do we identify with and acknowledge the value of the activity, but we find it consistent with our own values and interests.

“Autonomous extrinsic motivations share with intrinsic motivation the quality of being highly volitional, but differ primarily in that intrinsic motivation is based in interest and enjoyment—people do these behaviors because they find them engaging or even fun, whereas identified and integrated motivations are based on a sense of value—people view the activities as worthwhile, even if not enjoyable” (Ryan and Deci, 2020, p. 3).

**Figure 1:**
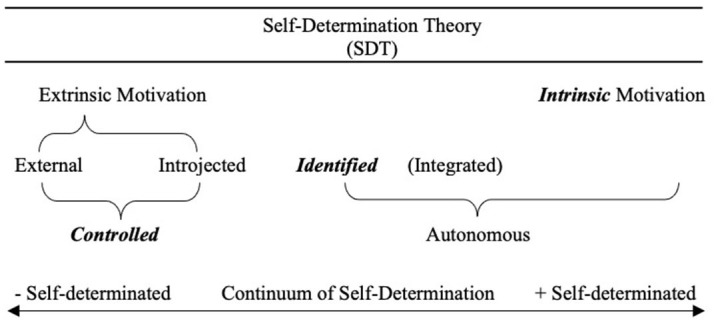
Motivation in Self-Determination Theory (Ryan and Deci, 2017, 2019). Note. Following previous studies (Guay et al., 2010), the three types of motivation considered in this work to assess motivation at early ages are highlighted in bold italics: Intrinsic motivation and identified motivation as autonomous and external and introjected motivation as controlled.

When SDT has been used at early ages, the self-determination continuum has been simplified by eliminating, on the one hand, integrated regulation, as it requires a formed identity that is difficult to find at such early ages (see Shahar et al., 2003; Guay et al., 2010) and, on the other hand, by merging into a single construct - *controlled regulation*- two types of extrinsic motivation: introjected and external (see Figure 1). The latter decision aims to reduce the number of items to which children have to respond and to adapt them to their abilities. This decision makes it possible to distinguish between behaviors derived from intrinsic or extrinsic motives, as well as to differentiate between the following extrinsic motives: seeking a reward or avoiding punishment, feelings of obligation or pride (controlled regulation) or perceived usefulness or personal importance (identified regulation).

The multidimensional framework proposed by SDT has received recent evidence corroborating the self-determination continuum (Howard et al., 2020; Bureau et al., 2022). The latest meta-analyses (Howard et al., 2020; Bureau et al., 2021) reveal that the different types of motivation (intrinsic and identified - more autonomous; external and introjected - more controlled) follow a simple correlation structure. The highest correlations would be between those motivations that are closer on the theoretical continuum, and the lowest correlations would be between those motivations that are further apart in the continuum. These data confirm the robustness of the theoretical framework proposed by SDT, validating its use in the study of academic motivation from an early age.

Research has identified distinct relationships between the types of motivation considered in this theory and academic performance (Guay et al., 2010; Garon-Carrier et al., 2016; Howard et al., 2021). For example, students with more autonomous (intrinsic and identified) types of motivation are known to be more persistent in solving tasks, more engaged, experience more positive feelings and perform better than students with controlled regulation (see Guay et al., 2008; Howard et al., 2021; Guay, 2022).

In order to assess these relationships, different instruments have been developed and validated [e.g.: Children's Academic Intrinsic Motivation Inventory, Gottfried, 1986 (from 4th grade of Primary School); Educational Motivation Scale, Núñez Alonso et al., 2006 (validated in university students); Intrinsic Motivation Inventory, Ryan, 1982 (validated in education from 5th grade of Primary School, Monteiro et al., 2015)], as well as others more specific to assess motivation in content domains such as reading [e.g., Reading Motivation Scale, Gomes and Boruchovitch, 2015 (from 3rd grade Primary); Reading Motivation Questionnaire, Wigfield and Guthrie, 1997; Schiefele and Schaffner, 2016 (for Secondary)], mathematics [e.g., Academic Motivation Toward Mathematics Scale, Lim and Chapman, 2015 (for upper secondary school)] or writing [e.g., Self-Regulation Questionnaire-Writing Motivation, De Smedt et al., 2020 (from Primary 3)].

Although there are studies that propose scales for evaluating motivation from an early age, they are often focused on only one concrete type of motivation [intrinsic; e.g., Young Children's Academic Intrinsic Motivation Inventory (YCAIMI), Gottfried, 2019], or on only one domain (reading, for example; Reading Motivation Scale, Gomes and Boruchovitch, 2015). In addition, researchers have shown an increased interest in exploring other issues (e.g., self-regulation) or motivational variables (e.g., personal beliefs such as self-efficacy) that require further cognitive development in learners. However, SDT allows the exploration of academic motivation at an earlier age, since it focuses attention on the different types of interest - intrinsic or extrinsic - that students may begin to develop toward specific content such as reading and mathematics. The problem is that there is a lack of instruments to assess all these aspects in younger populations. This scale developed by Guay et al. (2010) would allow to advance what has already been achieved by exploring various types of motivation - autonomous and controlled - in relation to various specific and elementary domains at the academic level - reading and mathematics, for example. This is very relevant in Spanish, where we cannot find a validated instrument of these characteristics and would greatly help advances in research. For example, the self-determination continuum (see Figure 1) considered in ESMS could facilitate not only the identification of the type of motivation most prevalent in students for specific domains, but also the adjustment of effort, in terms of situational support (Linnenbrink-Garcia and Patall, 2016), that educational agents should offer in and out of school. This possibility is provided by the Elementary School Motivation Scale -ESMS- (Guay et al., 2010) and, therefore, its validation in Spanish is an essential contribution to research.

Specifically, the ESMS assesses intrinsic, identified and controlled motivation in three specific content domains: reading, writing and mathematics. The original English version (Guay et al., 2010) with a sample of 426 students in the first three years of primary school showed acceptable reliability indices (α ranged between 0.70 and 0.90) for the different dimensions and a good fit for the first years of primary school (χ^2^/*df* = 1.99 and RMSEA = 0.048). Similar results were reported for the Russian version of the Elementary School Motivation Scale (ESMS-R) (Kanonire et al., 2020). Since its proposal, several works have used it (Pavalache-Ilie and Tîrdia, 2014; Garon-Carrier et al., 2016; Guay et al., 2017, 2019; De Sixte et al., 2020, among others).

The original proposal by Guay et al. (2010) used an earlier scale, the Academic Motivation Scale (AMS; Vallerand et al., 1992), as a reference. In a recent meta-analysis (Bureau et al., 2021), this scale was considered to be one of the most reliable and valid scales for exploring the motivational continuum according to SDT. This meta-analysis also shows that the use of this and other scales tend to be used from 4th/5th grade of primary school and for specific domains such as Physical Education (see Bureau et al., 2021).

The present study aims to validate the ESMS in a Spanish population at early ages. Specifically, (a) to assess internal consistency, (b) to confirm the factor structure of the model proposed by Guay et al. (2010), (c) to evaluate the self-determination continuum.

## Methods

### Participants

The sample was formed by 1,190 Spanish students in the first three stages of Primary Education (6–10 years old; *M* = 7.41 y SD = 0.94; see Table 1).

**Table 1:** Descriptives of the sample.

**Grade**	**Gender**		**Total (%)**
	**Female (%)**	**Male (%)**	
1	220 (50.90)	212 (49.10)	432 (36.30)
2	216 (49.90)	217 (50.10)	433 (36.40)
3	161 (49.50)	164 (50.50)	325 (27.30)
Total	597 (50.20)	593 (49.80)	1,190 (100)

A convenience sample was used, in accordance with that employed in the original study (Guay et al., 2010). The following inclusion criteria were considered: (a) to be regularly attending classes; (b) to not present any pathology that impedes questionnaire completion; (c) to have Spanish as mother tongue.

### Instrument

The Elementary School Motivation Scale (ESMS) was used (Guay et al., 2010), which is formed by 27 items structured in nine dimensions (see Table 2). The scale uses three items to assess each type of motivation (intrinsic, identified and controlled) for each specific content (reading, writing and mathematics). All items were scored using a 5 point Likert scale, being (1) “always no” and (5) “always yes.” Therefore, scores for each dimension fluctuated from 3 to 15 points.

**Table 2:** Items in each dimension.

**Dimension**	**Items**
Intrinsic reading motivation	L1, L2, L3
Identified reading motivation	L4, L5, L6
Controlled reading motivation	L7, L8, L9
Intrinsic mathematics motivation	M1, M2, M3
Identified mathematics motivation	M4, M5, M6
Controlled mathematics motivation	M7, M8, M9
Intrinsic writing motivation	E1, E2, E3
Identified writing motivation	E4, E5, E6
Controlled writing motivation	E7, E8, E9

**Figure 2:**
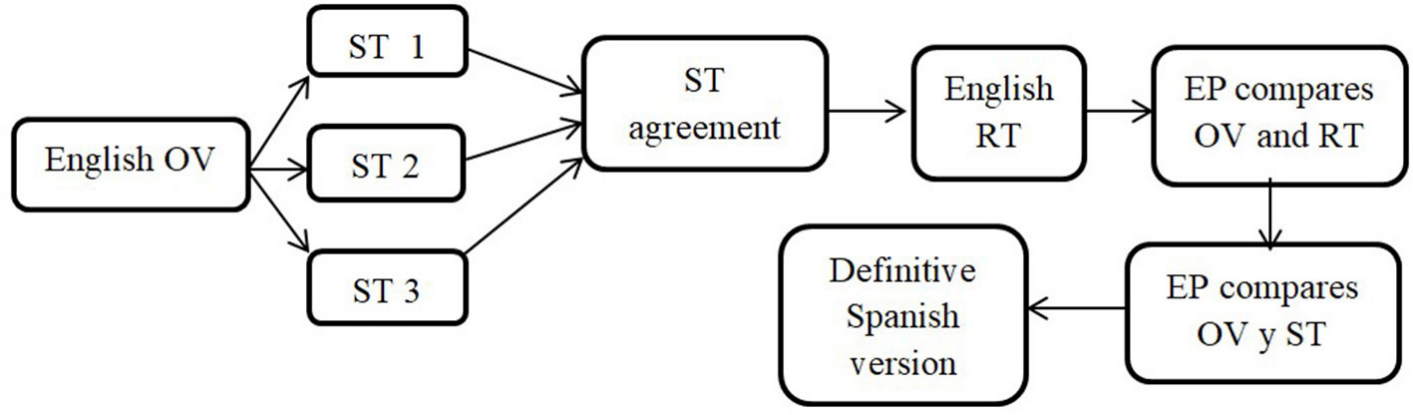
Scale translation process.

The guidelines proposed by the International Test Commission (2017) were followed to translate and validate the scale in Spanish, and consent was obtained from the author of the original scale. For the Spanish translation, the reverse translation strategy was chosen (Hambleton, 1996). A summary of the process can be seen in Figure 2. Three experts translated the items into Spanish and compared their individual translations until they reached an agreement. Then, a different group of experts translated those items into English and an expert panel compared them with the original ones to check their equivalence. Items were then assessed by experts in the motivation field to check the adequacy of the items to the construct they were referring to. Finally, the items in Spanish were revised to make sure they were easy to understand by the target population. The definitive version was administered in paper and was denominated Elementary School Motivation Scale-Español (ESMS-E; Appendix 1).

### Procedure

Different schools were contacted to explain the objective of the research and to ask for their collaboration. Those schools that voluntarily accepted to collaborate were asked to facilitate the distribution of an informed consent to the parents of the target students. Children whose parents filled out and signed the informed consent documents were assessed collectively in their classrooms by an expert. Students were assessed in a single session, using pencil and paper materials in their respective class groups.

Two pilot studies were performed. The first one using 91 students (50 in Year 1 of Primary Education, 41 in Year 2). This study showed that some improvements were necessary to facilitate comprehension in this population: emoticons were included for each Likert score (see Appendix 2) and each item should be read aloud by the researcher administering the scale. The second pilot study included these improvements with a sample of 440 students (146, 131, and 163 in Years 1, 2, and 3 months respectively). The comprehension of the scale was now accurate for the younger children and it was not interfering for the older ones.

### Data analysis

Internal consistency for the ESMS-E was assessed using Composite Reliability (CR). This index ranges from 0 to 1, and according to Hair et al. (2018), those values equal or higher than 0.7 are considered acceptable.

In order to analyse the model proposed by Guay et al. (2010) a Confirmatory Factor Analysis (CFA) was used. Since there was a lack of multivariate normality (as assessed by Mardia's coefficient), the polychoric correlation was used through the robust weighted least square mean and variance estimator (WLSMV), which is appropriate in these cases (Xia, 2016). In order to assess the goodness of fit of the model, researchers recommend several different indexes (Kline, 1998). Regarding global fit, one of the most relevant indexes for big samples is the standard chi-square (χ^2^/*df* ). Values lower than 2 are considered a very good fit and values between 3 and 5 are acceptable (Hair et al., 2018). To assess the residual matrix, the Root Mean Square Error of Approximation (RMSEA) was used. Values lower than 0.05 are considered very good and values between 0.05 and 0.08 are acceptable (Kline, 1998). To assess comparative fits, the Comparative Fit Index (CFI) and the Tucker–Lewis Index (TLI) were used. Values higher than 0.90 are considered acceptable in both indexes (Bentler, 1990).

The SDT continuum was assessed through correlations among the different types of motivation (intrinsic, identified and controlled) for each specific content (reading, writing and mathematics).

The statistical software used was the SPSS (v.26) and Mplus.

## Results

The descriptive statistics for each item and dimension in the ESMS-E can be seen in Table 3.

**Table 3:** Descriptive statistics for items and dimensions (*N* = 1,190).

**Dimension**	**Item**	**M**	**SD**	**Min**	**Max**
Intrinsic reading motivation	L1	4.131	1.184	1	5
	L2	3.955	1.238	1	5
	L3	3.534	1.406	1	5
	Total	11.620	2.918	3	15
Intrinsic mathematics motivation	M1	4.108	1.347	1	5
	M2	4.115	1.234	1	5
	M3	3.518	1.440	1	5
	Total	11.742	3.169	3	15
Intrinsic writing motivation	E1	4.094	1.261	1	5
	E2	3.951	1.231	1	5
	E3	3.816	1.358	1	5
	Total	11.861	3.058	3	15
Identified reading motivation	L4	4.418	0.976	1	5
	L5	4.029	1.208	1	5
	L6	4.646	0.843	1	5
	Total	13.094	2.142	3	15
Identified mathematics motivation	M4	4.513	0.888	1	5
	M5	4.229	1.111	1	5
	M6	4.479	0.924	1	5
	Total	13.222	2.160	3	15
Identified writing motivation	E4	4.230	1.100	1	5
	E5	4.085	1.149	1	5
	E6	4.587	0.847	1	5
	Total	12.902	2.346	3	15
Controlled reading motivation	L7	3.618	1.442	1	5
	L8	3.945	1.286	1	5
	L9	3.508	1.469	1	5
	Total	11.071	3.384	3	15
Controlled mathematics motivation	M7	3.856	1.387	1	5
	M8	3.971	1.279	1	5
	M9	3.656	1.413	1	5
	Total	11.48	3.323	3	15
Controlled writing motivation	E7	3.749	1.389	1	5
	E8	3.903	1.323	1	5
	E9	3.689	1.4426	1	5
	Total	11.342	3.455	3	15

Results show and average score for the dimensions that range from 11.071 to 13.222, indicating high levels of motivation irrespective of the type and the domain. However, results seem coherent across all domains (reading, mathematics and writing): the lowest values are obtained in controlled motivation in each domain (Mreading = 11.071; Mwriting = 11.342; Mmathematics = 11.483), whereas the highest scores are for identified motivation (Mmathematics = 13.222; Mreading = 13.094; Mwriting = 12.902).

### Reliability

Internal consistency for the nine dimensions was assessed through composite reliability. As shown in Table 4, all indexes are over the acceptable level (0.7), confirming an appropriate reliability.

**Table 4:** Reliability statistics.

**Dimension**	**Composite**	**Cronbach's alpha**
	**reliability**	**(original**
	**(ESMS-E)**	**ESMS)**
Intrinsic reading motivation	0.816	0.76
Identified reading motivation	0.701	0.70
Controlled reading motivation	0.869	0.73
Intrinsic mathematics motivation	0.896	0.80
Identified mathematics motivation	0.797	0.81
Controlled mathematics motivation	0.885	0.90
Intrinsic writing motivation	0.867	0.78
Identified writing motivation	0.809	0.79
Controlled writing motivation	0.901	0.80

### Confirmatory Factor Analysis

**Figure 3:**
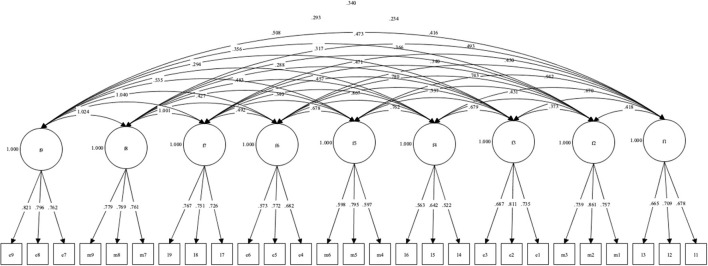
Proposed model for the ESMS-E with weights and correlations between errors.

After checking a lack of multivariate normality (Mardia's coefficient = 121.33, *p* < 0.01) robust estimators were used (WLSMV) to assess the model. The CFA assessed the factorial structure of the nine dimensions. Results showed reasonable evidence for the factorial structure of the ESMS-E, confirming that the factors correspond to the original ESMS (see Table 5 and Figure 3).

**Table 5:** Goodness of fit model comparison.

**Model**	***N***	**χ^2^**	***df***	***p*-Values**	**χ^2^/*df***	**RMSEA**	**CI 90%**		**CFI**	**TLI**
							**Lower**	**Upper**		
Proposed ESMS-E	1,190	1.711	288	0.000	5.941	0.064	0.061	0.067	0.929	0.913
Original ESMS	425	518.59	261		1.99	0.048	0.042	0.054		

Table 6 shows how factorial weights for each item in all nine dimensions range from 0.522 to 0.861. Since all values are over 0.4, all of them make a significant contribution to the factor (Morales, 2000). The strongest items in the model are M2 (*R*^2^ = 0.741) and E9 (*R*^2^ = 0.673), explaining more than 65% of the variance for the corresponding factor. The weakest items are L4 (*R*^2^ = 0.273) and L6 (*R*^2^ = 0.317), explaining around 30% of the variance. However, all items showed a shared variance to the factor that was statistically significant (*p* < 0.01).

**Table 6:** Factorial weights and shared variance between item and factor.

**Factor**		**Item**	**Factorial weight**	***R* ^2^**
1	Intrinsic reading motivation	L1	0.678^*^ (0.026)	0.460^*^ (0.035)
		L2	0.709^*^ (0.025)	0.503^*^ (0.035)
		L3	0.665^*^ (0.028)	0.442^*^ (0.038)
2	Intrinsic mathematics motivation	M1	0.757^*^ (0.021)	0.573^*^ (0.033)
		M2	0.861^*^ (0.019)	0.741^*^ (0.033)
		M3	0.739^*^ (0.027)	0.546^*^ (0.040)
3	Intrinsic writing motivation	E1	0.735^*^ (0.021)	0.540^*^ (0.030)
		E2	0.811^*^ (0.021)	0.657^*^ (0.034)
		E3	0.687^*^ (0.025)	0.472^*^ (0.035)
4	Identified reading motivation	L4	0.522^*^ (0.031)	0.273^*^ (0.032)
		L5	0.642^*^ (0.029)	0.412^*^ (0.037)
		L6	0.563^*^ (0.036)	0.317^*^ (0.041)
5	Identified mathematics motivation	M4	0.597^*^ (0.030)	0.357^*^ (0.036)
		M5	0.795^*^ (0.029)	0.632^*^ (0.045)
		M6	0.598^*^ (0.031)	0.358^*^ (0.037)
6	Identified writing motivation	E4	0.682^*^ (0.025)	0.465^*^ (0.034)
		E5	0.772^*^ (0.022)	0.596^*^ (0.034)
		E6	0.573^*^ (0.032)	0.328^*^ (0.037)
7	Controlled reading motivation	L7	0.726^*^ (0.017)	0.527^*^ (0.025)
		L8	0.751^*^ (0.017)	0.563^*^ (0.025)
		L9	0.767^*^ (0.015)	0.589^*^ (0.023)
8	Controlled mathematics motivation	M7	0.761^*^ (0.016)	0.580^*^ (0.025)
		M8	0.769^*^ (0.015)	0.592^*^ (0.023)
		M9	0.779^*^ (0.015)	0.607^*^ (0.023)
9	Controlled writing motivation	E7	0.762^*^ (0.015)	0.581^*^ (0.023)
		E8	0.796^*^ (0.014)	0.634^*^ (0.022)
		E9	0.821^*^ (0.013)	0.673^*^ (0.021)

* p < 0.01 (all values two-tailed).

Standard errors are showed in brackets.

### Correlations

Table 7 shows the correlations between factors to assess the existence of the continuum proposed by SDT. These correlations suggest that the continuum is valid in Spanish population for the target age range, since correlations between contiguous types of motivation (for example, in reading *M*_intrinsic_-*M*_identified_ *r* = 0.942, *p* < 0.01 or *M*_identified_-*M*_controlled_ *r* = 0.457, *p* < 0.01) are stronger than correlations between distant types of motivation (again with reading Motivation_intrinsic − controlled_ *r* = 0.416, *p* < 0.01). This same continuum can be observed for mathematics (Motivation_intrinsic − identified_ *r* = 0.743, *p* < 0.01, Motivation_identified − controlled_ *r* = 0.483, *p* < 0.01, Motivation_intrinsic − controlled_ *r* = 0.473, *p* < 0.01), and for writing (Motivation_intrinsic − identified_ *r* = 0.780, *p* < 0.01, Motivation_identified − controlled_ *r* = 0.535, *p* < 0.01, Motivation_intrinsic − controlled_ *r* = 0.508, *p* < 0.01).

**Table 7:** Correlations between factors.

**Factor**	**Intrinsic**	**Identified**	**Controlled**
**Reading**
Intrinsic	–		
Identified	0.942^*^ (0.038)	–	
Controlled	0.416^*^ (0.039)	0.457^*^ (0.043)	–
**Mathematics**
Intrinsic	–		
Identified	0.743^*^ (0.029)	–	
Controlled	0.473^*^ (0.033)	0.483^*^ (0.037)	–
**Writing**
Intrinsic	–		
Identified	0.780^*^ (0.028)	–	
Controlled	0.508^*^ (0.032)	0.535^*^ (0.034)	–

* p < 0.01 (all values two-tailed).

Standard errors are showed in brackets.

## Discussion and conclusion

The ESMS is an instrument that has been validated in English or Russian, and it has been widely used in many countries, such as Canada, Romania or Russia (e.g., Pavalache-Ilie and Tîrdia, 2014; Garon-Carrier et al., 2016; Guay et al., 2019; Kanonire et al., 2020). The aim of the present study was to validate the ESMS (developed by Guay et al., 2010) in Spanish population to be used in Primary Education. This is the first study that attempts such validation in Spain.

In general, results showed acceptable psychometric properties of the ESMS-E for the target population. Regarding the first objective, ESMS-E shows good internal consistency and the instrument is consistent with the original one (Guay et al., 2010). Furthermore, the reliability indexes obtained in the present study are higher than those obtained in the original instrument, except for two dimensions: Identified mathematics motivation and controlled mathematics motivation. A potential explanation of these differences might be the different analyses performed. The original instrument used Cronbach's alpha to assess the internal consistency of the nine dimensions. However, the fact that the scale uses ordinal scores might cause issues with Cronbach's alpha, which might explain why they obtain lower values than the ones presented here, using a more appropriate test for this type of data (composite reliability).

Regarding the second objective, the Confirmatory Factor Analysis corroborated the original theoretical structure suggested by Guay et al. (2010). Results for the ESMS-E support the nine original factors in the ESMS, with acceptable fit index scores that confirm its validity (Hu and Bentler, 1999). The analyzed dimensions fit the different types of motivation suggested by the SDT (Ryan and Deci, 2017, 2019; Howard et al., 2020, intrinsic, identified, controlled) in all three specific domains that the instrument assesses (reading, writing and mathematics).

Regarding the third objective, correlations between dimensions proved the presence of a continuum as proposed by the theory: highest correlations between contiguous motivations and lower correlations between distant ones (Howard et al., 2020; Bureau et al., 2022). These results corroborate the importance of considering the different types of motivation that are present in early ages for each specific domain.

In conclusion, the ESMS-E is a valid instrument to assess motivation in Spanish students aged 6–10 years old toward different content areas. This is of key importance since motivation is a recognized predictor of academic achievement in different areas (e.g., Miñano and Castejón, 2011; Mercader et al., 2017; De Sixte et al., 2020). Furthermore, initial motivation to learn a specific content not only benefits immediate performance but long-term achievement too (e.g., Reimann et al., 2013; Mercader et al., 2017). It might prove of great importance in helping teacher and education professionals to design better individualized interventions, since motivation is a decisive factor in the efficacy of the interventions on learning difficulties (Ise and Schulte-Körne, 2013; Dubois et al., 2022) and that teachers who support student autonomy can foster autonomous motivation (Guay, 2022). From a prevention point of view, assessing the different types of motivation in early ages would allow the design and implementation of programs aimed at reducing controlled motivation, keeping in mind that such programs “should take place even before children enter elementary school” (Guay et al., 2010, p. 730). Also, it would allow identifying those students with lower self-determined behaviors, since they have higher probabilities to quit their studies or to show lower well-being (Howard et al., 2021). In sum, knowing the motives that drive students at early ages would improve the quality of the learning process.

The strengths of the present study are the great sample size, the use of a robust methodology appropriate to ordinal items and lack of normality (Xia, 2016) and the differentiation of the different types of motivation suggested by the SDT (Ryan and Deci, 2017, 2019; Howard et al., 2020), including three specific domains (reading, writing and mathematics) in the same instrument. Also, this is the first study to validate the ESMS in Spanish population for students in the first 3 years of Primary Education (ages 6–10).

Despite these strengths, the present study has some limitations, such as not using a probabilistic sampling method that could facilitate an easier generalization of the instrument. Also, it is a transversal study. Although this is a common practice in this kind of research, future studies should explore this topic using longitudinal designs to corroborate the suggested structure and reliability of the instrument. Future lines of research that can derive from the results obtained in this work will make it possible to answer questions such as: What is known about the type of motivation displayed by students in the first years of schooling?; Is it possible to calibrate the types of motivation according to the specific content -reading, writing and mathematics-?; What is their relationship with performance in each domain?; What role do teachers and families play as educational agents of reference in promoting each type of motivation?; These are some of the questions that could guide future lines of research.

To sum up, the Spanish version of the ESMS (ESMS-E) is a reliable instrument that allows the assessment of motivation at early ages, considering different types of motivation (intrinsic, identified and controlled) and three specific domains (reading, writing and mathematics). Therefore, it can be incorporated to the educational practice to assess motivation as proposed by the SDT, to explore its development and to analyse its impact on different specific contents.

## Data availability statement

The original contributions presented in the study are included in the Supplementary material, further inquiries can be directed to the corresponding author/s.

## Ethics statement

The studies involving human participants were reviewed and approved by Project PSI2015-66802-P del Ministerio de Economía y Competitividad. Written informed consent to participate in this study was provided by the participants' legal guardian/next of kin.

## Author contributions

MR and RD implemented the study and drafted the manuscript. MR analyzed and interpreted the data. RD, ÁJ, and JR reviewed the study and performed substantial suggestions. All authors contributed to data collection, read, and approved the final manuscript.

## Conflict of interest

The authors declare that the research was conducted in the absence of any commercial or financial relationships that could be construed as a potential conflict of interest.

## Publisher's note

All claims expressed in this article are solely those of the authors and do not necessarily represent those of their affiliated organizations, or those of the publisher, the editors and the reviewers. Any product that may be evaluated in this article, or claim that may be made by its manufacturer, is not guaranteed or endorsed by the publisher.
