# Supplementary material for: Academic motivation at early ages: Spanish validation of the Elementary School Motivation Scale (ESMS-E)
Source: Front Psychol. 2022 Dec 7;13:980434. doi: 10.3389/fpsyg.2022.980434 (PMC9769706; doi:10.3389/fpsyg.2022.980434)
Supplement: Supplementary file 1 [file Presentation_1.pdf]

Appendix 1

ESMS-E

**Motivación intrínseca lectura** [Intrinsic Reading Motivation]	
L1. Me gusta leer [I like reading]	
L2. La lectura me gusta mucho [Reading interests me a lot]	
L3. Leo incluso cuando no tengo que hacerlo [I read even when I don't have to]	
**Motivación identificada lectura** [Identified Reading Motivation]	
L4. Puedo aprender muchas cosas útiles leyendo [I can learn many useful things by reading]	
L5. Elijo leer para aprender muchas cosas [I choose to read to learn many things]	
L6. En la vida es importante leer [In life, it's important to learn how to read]	
**Motivación controlada lectura** [Controlled Reading Motivation]	
L7. Leo para conseguir una recompensa agradable [I read to get a nice reward]	
L8. Leo para darle el gusto a mis padres o a mi profesor [I read to please my parents or my teacher]	
L9. Leo para demostrar a los demás lo bueno que soy [I read to show others how good I am]	
**Motivación intrínseca matemáticas** [Intrinsic Mathematics Motivation]	
M1. Me gustan las matemáticas [I like maths]	
M2. Las matemáticas me interesan mucho [Maths interests me a lot]	
M3. Estudio matemáticas incluso si no tengo que hacerlo [I do maths even when I don't have to]	
**Motivación identificada matemáticas** [Identified Mathematics Motivation]	
M4. Puedo aprender muchas cosas útiles a través de las matemáticas [I can learn many useful things by doing maths]	
M5. Elijo practicar matemáticas para aprender muchas cosas [I choose to do maths to learn many things]	
M6. En la vida, es importante aprender cómo practicar las matemáticas [In life, it's important to learn how to do maths]	
**Motivación controlada matemáticas** [Controlled Mathematics Motivation]	
M7. estudio matemáticas para conseguir una recompensa agradable [I do maths to get a nice reward]	
M8. Estudio matemáticas para darle el gusto a mis padres o a mi profesor [I do maths to please my parents or my teacher]	
M9. Estudio matemáticas para demostrar a los demás lo bueno que soy [I do maths to show others how good I am]	
**Motivación intrínseca escritura** [Intrinsic Writing Motivation]	
E1. Me gusta escribir [I like writing]	
E2. La escritura me gusta mucho [Writing interests me a lot]	
E3. Escribo incluso cuando no tengo que hacerlo [I write even when I don't have to]	
**Motivación identificada escritura** [Identified Writing Motivation]	
E4. Puedo aprender muchas cosas útiles escribiendo [I can learn many useful things by writing]	
E5. Elijo escribir para aprender muchas cosas [I choose to write to learn many things]	
E6. En la vida, es importante aprender a escribir [In life, it's important to learn how to write]	
**Motivación controlada escritura** [Controlled Writing Motivation]	
E7. Escribo para conseguir una recompensa agradable [I write to get a nice reward]	
E8. Escribo para darle el gusto a mis padres o a mi profesor [I write to please my parents or my teacher]	
E9. Escribo para demostrar a los demás lo bueno que soy [I write to show others how good I am]	

Note: Original items (Guay et al., 2010) are in brackets

Appendix 2

Presentation example with emoticons

1. Me gusta leer [I like reading]

No siempre	A veces no	No lo sé	A veces sí	Siempre
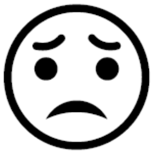	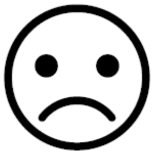	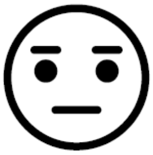	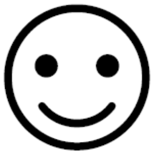	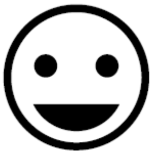

Note: "always no", "sometimes no", "I don´t Know", "sometimes yes", "always yes".
